# Selection of a Suitable Disc Bioassay for the Screening of Anti-Tumor Molecules

**Published:** 2013-12

**Authors:** Fatma Trigui, Pascal Pigeon, Karim Jalleli, Siden Top, Sami Aifa, Mehdi El Arbi

**Affiliations:** 1 Centre de Biotechnologie de Sfax (Université de Sfax). Route de Sidi Mansour Km 6, BP 1177, 3018 Sfax, Tunisia;; 2 Chimie ParisTech (Ecole Nationale Supérieure de Chimie de Paris), Laboratoire Charles Friedel, UMR CNRS 7223, 11 rue Pierre et Marie Curie, 75231 Paris Cedex 05, France

**Keywords:** *Agrobacterium tumefaciens*, antitumor activity, beet discs, bioassay, chemical synthesis, organometalics

## Abstract

The crown gall induced in potato discs by *Agrobacterium tumefaciens* is becoming largely utilised in screening anti-tumor agents. The present work is showing that beet discs are more adequate for the anti-tumor screening test. In fact, maximal tumor induction was observed on beet discs (87.5%), followed by carrot discs (75%) and potato discs (68.5%). Beet discs present the most sensibility to crown gall disease with a fast expression of symptoms and more visible galls without any staining need. The beet discs bioassay was carried out by using some synthesized organometallics known for their antitumor activity in mammalian cells. We found significant crown gall inhibition (20.7% to 40.55%) of the tested compounds. Overall results supported that beet bioassay might be a potential prescreen system of anti-tumor molecules in mammalian cells.

## INTRODUCTION

Bioassay methods offer special advantages in establishing the biological purposes (antitumor, antibacterial, antioxidant and phytotoxic properties. etc.). Bioassay is the preliminary step in drug discovery which allow the screening of biological and synthetic bioactive compounds (1). Potato disc assay was shown to be useful for checking known and novel antitumor molecules’ properties. This bioassay is based on *Agrobacterium tumefaciens* infection on potato disc (2).The validity for the use of such assay is that the tumorogenic mechanism initiated in plant tissues by *A. tumefaciens* is in many ways similar to that of animals (3). In fact, Kempf, *et al.*, (4) showed that *Bartonella henselae*, a bacterium causing tumor in human shares a similar pathogenicity strategy, with plant pathogens *A. tumefaciens*. Several studies are finding numerous and significant areas of similarity among the mechanisms used by bacterial pathogens of plants and humans. These similarities include the use of common toxins, secretion system, adhesion mechanism, invasion and regulation (5).

This method is used during the last 15 years and it appears to be adaptable to the purpose of standardization or quality control of bioactive compounds (6). It has been shown that the inhibition ability of crown gall formation by *A. tumefaciens* on potato discs and subsequent growth was in good correlation with compounds and extracts, which are statistically much more predictive of *in vivo* and *in vitro* anti-leukemic activity (1, 7-9). Several studies have demonstrated that Podophyllin, Taxol, Camptothecin, Vincristine and Vinblastine have shown significant tumor inhibition in the 3PS leukemic mouse assay. These compounds have already been tested for their inhibitory action on tumor formation by potato disc tumor assay (7, 10-13).

*A. tumefaciens,* is a Gram-negative soil borne bacterium, rod–shaped and virulent that is the causative agent of Crown Gall Disease. Crown Gall is a neoplastic disease in which a mass of tissue bulging from stems and roots of woody and herbaceous plants is produced. The tumor masses could be spongy or hard, with or without a deleterious effect on the plant. The produced tumor is histologically similar to animal or human ones. The process of tumor induction by Ti-plasmid is the result of cell proliferation and blocking of apoptosis like in animal or human cancer cells (14). As a consequence, it was proposed to adopt the crown gall tumor (potato disc) assay as a prescreen for antitumor activity (14-15). Although aseptic technique is required, the methodology is simple and can be performed with minimal technical training. The authors have since used this assay to detect and isolate novel antitumor compounds.

The antitumor potato disc assay was shown to be sensitive for variable chemicals that interfere with cell cycle and have different modes of action (9, 16). This simple test that needs aseptic conditions has allowed the detection and isolation of many anti tumor compounds from plant microbial or biomolecules that were confirmed by *in vivo* animal tumor inhibition (6).

Considering the continuous demand for antitumor drugs in the area of tumor/cancer inhibition assay, the present study was undertaken to improve the screening system based on potato by the use of other vegetables such as beet, radish and carrot disc bioassay and to evaluate the activity of known antitumor organometallic molecules in the more appropriate vegetable disc system.

## MATERIAL AND METHODS

### Phytopathogenicity test

Phytopathogenicity tests were done using beet, radish, carrot (1, 17-18) and potato (19) disc bioassays. The strain C58 of *A. tumefaciens* was used for the tumor induction.

### Potato, carrot, radish and beet disc bioassay

*A. tumefaciens *strains were cultured on Luria Bertani (LB) agar medium. A single colony was transferred into LB broth medium and incubated at 30°C for 24 h. Beets (*Beta vulgaris* L.), carrots (*Daucas carota* L.)*, *potatoes (*Solanum tuberosum* L.) and radishes (*Raphanus sativus* L.) were disinfested by scrubbing under running water with a brush, then immersed in 2% Clorox for 5 min. Potato, carrot, radish and beet discs (5 mm x 8 mm) were made with cork borer and immersed in 2% Clorox for 30 min. Each disc was rinsed thrice in autoclaved distilled water for 15 min. After rinsing, the discs were removed from the distilled water, blotted on sterile paper towels. Sixteen discs were placed on Petri plates containing autoclaved agar medium (2%). Suspensions of *A. tumefaciens* on LB broth medium were standardized to 10^7^ CFU/ml as determined by an absorbance value of 0.96 ± 0.02 at 600 nm. Each disc was overlaid with 50 μL of bacterial suspension. Petri plates were sealed by parafilm and incubated at room temperature (25-30°C). Ten replications were used and experiment was repeated at least twice.

Carrot discs were checked after 10 days for young galls developing from meristematic tissue around vascular system. Beet discs were checked also after 10 days of tumors development on the entire disc surface. After 21 days, potato discs were stained with Lugol’s solution (10% KI + 5% I_2_) and tumors were counted under dissecting microscope (19). Lugol’s reagent stains the starch in the potato tissue a dark blue to dark brown colour, but the tumors produced by *A. tumefaciens* will not take up the stain, and appear creamy to orange (6).

### Chemical compounds

**Figure 1:**
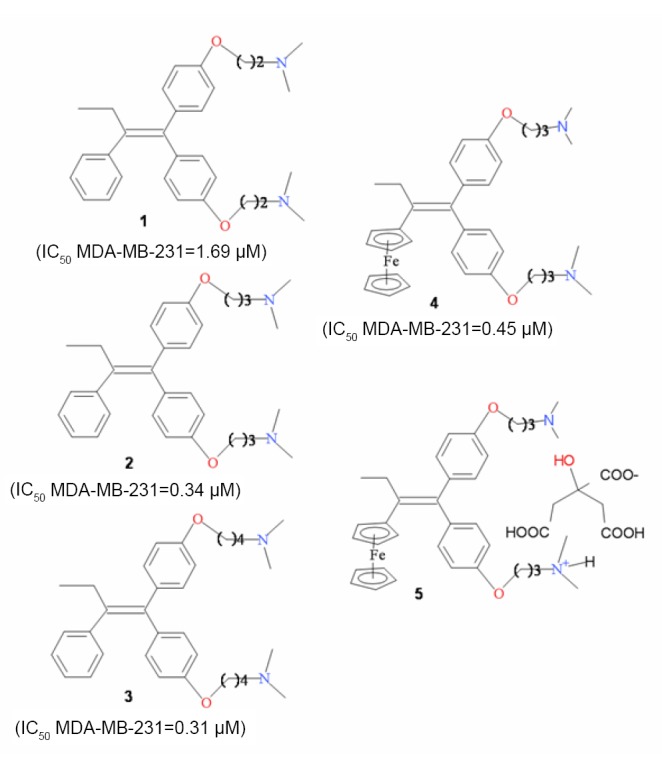
Chemical structure of the tested compounds.

The compounds 1,1-Bis[4-(2-dimethylaminoethoxy)phenyl]-2-phenyl-but-1-ene [1] (20), 1,1-Bis[4-(3-dimethylaminopropoxy)phenyl]-2-phenyl-but-1-ene [2] (21), 1,1-Bis[4-(3-dimethylaminopropoxy)phenyl]-2-ferrocenyl-but-1-ene [4] (22), 1-bis[4-(3-dimethylamoniumpropoxy)phenyl]-2-ferrocenyl-but-1-ene citrate [5] (22), were synthesized as described in the above references and have already been reported for their antiproliferative activity on breast cancer cells. 1, 1-Bis [4-(4-dimethylaminobutoxy) phenyl]-2-phenyl-but-1-ene [3] is a newly synthesized compound (Figure 1).

### Chemical experimental Section

THF and diethyl ether were obtained by distillation from sodium/benzophenone. Thin layer chromatography was performed on silica gel 60GF254. Column Chromatography purifications were performed by using silica gel with appropriate eluent. ^1^H and ^13^C NMR spectra were recorded on a 300 MHz Bruker spectrometer. Mass spectrometry was performed with a Nermag R 10–10C spectrometer.

### 1,1-Bis[4-(4-bromobutoxy)phenyl]-2-phenyl-but-1-ene 7

Compound **6** (2.531 g, 8 mmoles), cesium carbonate (5.213 g, 16 mmoles), and potassium carbonate (11.057 g, 80 mmoles) where stirred in 100 ml of acetone, then 1,4-dibromobutane (8.637 g, 4.78 ml, 40 mmoles) was added. The mixture was refluxed overnight, cooled and evaporated. The residue was dissolved in a mixture of dichloromethane and water and decanted. After drying on magnesium sulfate, the solution was evaporated and the residue was chromatographed on silica gel column with a cyclohexane/ethyl acetate 1/1 solution as the eluent to yield product **7** in 86% yield. The product was utilized in the next step without further purification. ^1^H NMR (CDCl_3_) : δ 0.94 (t, J=7.5 Hz, 3 H, CH_3_), 1.79-2.17 (m, 8 H, CH_2_-CH_2_), 2.50 (q, J=7.5 Hz, 2 H, CH_2_), 3.45 (t, J=6.5 Hz, 2 H, CH_2_Br), 3.52 (t, J=6.5 Hz, 2 H, CH_2_Br), 3.86 (t, J=6.0 Hz, 2 H, CH_2_O), 4.02 (t, J=6.0 Hz, 2 H, CH_2_O), 6.54 (d, J=8.8 Hz, 2 H, C_6_H_4_), 6.78 (d, J=8.8 Hz, 2 H, C_6_H_4_), 6.88 (d, J=8.8 Hz, 2 H, C_6_H_4_), 7.07-7.22 (m, 7 H, C_6_H_5_+C_6_H_4_). ^13^C NMR (CDCl_3_) : δ 13.7 (CH_3_), 27.9 (CH_2_), 28.0 (CH_2_), 29.0 (CH_2_), 29.5 (CH_2_), 29.6 (CH_2_), 33.5 (2 CH_2_), 66.5 (CH_2_), 66.7 (CH_2_), 113.2 (2 CH C_6_H_4_), 113.9 (2 CH C_6_H_4_), 125.9 (CH C_6_H_5_), 127.9 (2 CH_arom_), 129.7 (2 CH_arom_), 130.6 (2 CH_arom_), 132.0 (2 CH_arom_), 135.9 (C), 136.4 (C), 137.8 (C), 141.0 (C), 142.6 (C), 156.7 (C), 157.6 (C). IR (KBr, ν cm^-1^): 3033, 2959, 2928, 2870 (CH, CH_2_, CH_3_). MS (EI, 70 eV) m/z: 584 [M]^+.^, 504 [M-HBr]^+.^.

### 1, 1-Bis [4-(4-dimethylaminobutoxy) phenyl]-2-phenyl-but-1-ene 3

In a pressure tube, compound **7** was dissolved in 2.0 M dimethylamine solution in methanol. The reaction mixture was heated to 60°C under stirring for 1 day. The mixture was concentrated under reduced pressure. Dichloromethane was added and the solution was washed with a 1 M sodium hydroxide solution, then with water. After decantation, the organic layer was dried on magnesium sulfate and concentrated under reduced pressure. The crude mixture was purified by flash column chromatography (Acetone/Et3N: 10/1) to give pure compound 3 in a 69% yield. ^1^H NMR (CDCl_3_) : δ 0.92 (t, J=7.4 Hz, 3 H, CH_3_), 1.50-1.88 (m, 8 H, 2 CH_2_-CH_2_), 2.20 (s, 6 H, NMe_2_), 2.24 (s, 6 H, NMe_2_), 2.27 (t, J=7.4 Hz, 2 H, CH_2_N), 2.33 (t, J=7.4 Hz, 2 H, CH_2_N), 2.48 (q, J=7.4 Hz, 2 H, CH_2_), 3.83 (t, J=6.2 Hz, 2 H, CH_2_O), 3.99 (t, J=6.2 Hz, 2 H, CH_2_O), 6.52 (d, J=8.8 Hz, 2 H, C_6_H_4_), 6.75 (d, J=8.8 Hz, 2 H, C_6_H_4_), 6.86 (d, J=8.8 Hz, 2 H, C_6_H_4_), 7.05-7.20 (m, 7 H, C_6_H_5_+C_6_H_4_). ^13^C NMR (CDCl_3_) : δ 14.0 (CH_3_), 24.6 (CH_2_), 24.7 (CH_2_), 27.5 (CH_2_), 27.6 (CH_2_), 29.4 (CH_2_), 45.8 (NMe_2_), 45.9 (NMe_2_), 59.8 (2 CH_2_), 67.7 (CH_2_), 68.0 (CH_2_), 113.5 (2 CH C_6_H_4_), 114.3 (2 CH C_6_H_4_), 126.2 (CH C_6_H_5_), 128.1 (2 CH_arom_), 130.0 (2 CH_arom_), 130.9 (2 CH_arom_), 132.2 (2 CH_arom_), 136.0 (C), 136.5 (C), 138.2 (C), 141.1 (C), 143.0 (C), 157.3 (C), 158.1 (C). IR (KBr, ν cm^-1^): 3035, 2945, 2869, 2813, 2762 (CH, CH_2_, CH_3_).

### Minimal inhibitory concentration

All synthesized compounds were tested against preliminary *A. tumefaciens* using microplate dilution method. Minimal inhibitory concentrations (MICs) of organic or organometallic molecules were determined according the National Committee for Clinical Laboratory Standard (NCCLS, 2002). The test was performed in sterile 96-well microplates. The inhibitory activity of synthesized compounds was properly prepared and transferred to each microplate well in order to obtain a twofold serial dilution of the original sample. The compounds were dissolved in dimethylsulfoxide (DMSO) or distilled water depending on their solubility. Serial twofold dilutions of each sample to be evaluated were made to yield volumes of 100 µl/well with final concentrations ranging from 2.54 × 10^-4^ M to 1.98 × 10^-6^ M. 100 µL of bacteria suspension with a concentration of 10^7^ CFU/ml were added to each well. Negative control wells contained bacteria only in LB broth medium. After incubation at 30°C for 16 h, the minimal inhibitory concentrations (MICs) were recorded as the lowest concentration of compound in the medium that showed no microbial growth by determining of the optical density (OD) at 620 nm. The OD was measured with a Biohrom microplate reader. Solvent, media and positive growth controls were also run simultaneously. The inhibitory activity of the tested compounds was calculated according to the following formula:

IA(%)=100-100(OD620(x)/OD620(i))

where (x) is the microbial culture containing the inhibitor and (i) is the microbial culture without inhibitor (23).

### Viability test of *A. tumefaciens* strains

Successful crown gall formation on a potato, radish, carrot and beet disc surface during an antitumor study is dependent on viable *A. tumefaciens*. It became necessary to avoid the lethal concentrations against *A. tumefaciens* (2, 19). Bacterial viability was determined by incubating each drug with 10^7^ CFU/ml of bacterial suspension. At 30 and 60 min of inoculation, 10 μl of each solution was removed and plated on LB plates, and incubated for 24 h. Bacterial growth was evidenced by growth across the plates.

### Antitumor Activity

For antitumor activity, the crown gall tumor inhibition assay (beet disc assay) was performed for compounds **2**, **4** and **5** using *A. tumefaciens* (C58). Numbers of tumors per disc were counted and the percentage of tumor inhibition was calculated using the following formula (15).

Percentage inhibition = 100 - Number of tumor with sample / Number of tumor with control × 100.

More than 20% tumor inhibition was considered as significant (8). Ten replications were used for each treatment and experiment was repeated twice.

## RESULTS

### Chemical experimental Section

**Figure 2:**
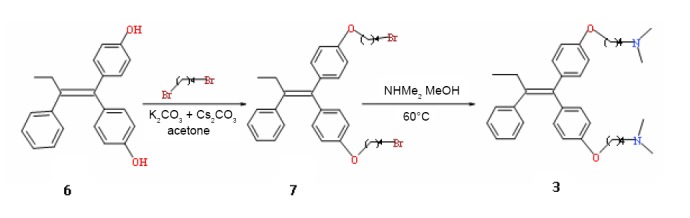
Chemical synthesis of compound 3.

1, 1-Bis[4-(4-dimethylaminobutoxy)phenyl]-2-phenyl-but-1-ene [3] was synthesized starting from biphenol **6** (23). First, compound **6** was converted into the dibrominated compound **7** by action of 1,4-dibromobutane with a cesium carbonate/potassium carbonate mixture as the bases in refluxing dry acetone in an 86% yield. This latter compound was then converted into the diamine compound **3** by action of dimethylamine in methanol at 60°C in a 69% yield (Figure 2).

### Phytopathogenicity test

The inoculation test on beet discs contained in Petri dishes gives the highest infection level and the fastest (8 days) followed by carrot, potato and radish discs.

**Figure 3:**
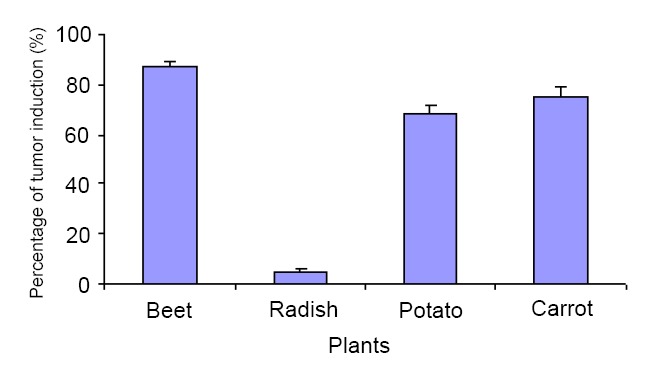
Percentage of tumor induction in Beet, Radish, Potato and Carrot discs.

The percentages of tumor induction were 87.5%, 75% and 68.5% for beet, carrot and potato discs, respectively (Figure 3). These percentages make them good candidates for determining the antitumor potentials of screened compounds. In the other hand the weak percentages of tumor induction of radish discs is not in favour of such application.

**Figure 4:**
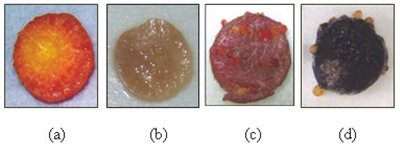
Phytopathogenicity test on different discs: (a) carrot (b) radish (c) beet (d) potato.

Moreover, the crown gall formation was observed after 8 days for both beet and carrot discs without any staining need. Meanwhile, the potato discs displayed the galls in 21 days after lugol staining. As a consequence the potato discs have a high risk of contamination and cost more for bioassay tests. The same conclusion was observed for radish discs with a higher risk of contamination which oblige the repetition of the test many times to get results (Figure 4).

These results allow selecting beet discs as the most sensitive to crown gall disease with a fast expression of symptoms, more visible galls without the need of any staining and homogeneous distributed galls on the entire surface of the disc. In fact, enumeration on carrot discs seems to be more difficult since the development of galls is limited in size and in space (around the vascular system) and originated from meristematic tissue taking its colour.

### Antibacterial Assay

The tested compounds used in this study are organic or organometallic with different length of the amine chain. Stock solutions of the tested products were prepared in DMSO or water, depending on their solubility. We tested all synthesized compounds against *A. tumefaciens* to estimate the effect of the amine chain length and the conversion into salts of citric acid on the antimicrobial activities in order to select the more appropriate products for the antitumor activity test.

**Figure 5:**
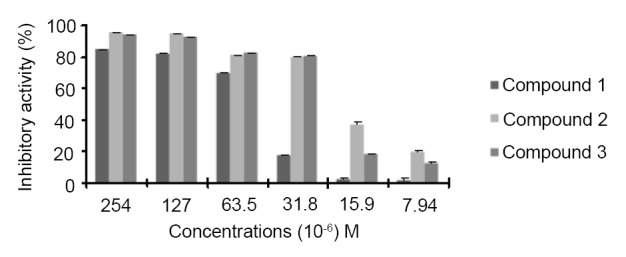
Antibacterial activity of compounds 1, 2 and 3 against *A.tumefaciens*.

To evaluate the impact of the amine chain length on the antibacterial activity, organic compounds **1**, **2** and **3**, having an amino chain of **2**, **3** and **4** carbons, respectively, were selected for the test with A. *tumefaciens*. At a concentration of 254 × 10^-6^ M *A. tumefaciens* was sensitive to the three compounds. Within these compounds, the compound with 3 carbons is more potent since the highest inhibitory activity was observed with compound **2** which inhibited bacterial growth at all concentrations (Figure 5).

**Figure 6:**
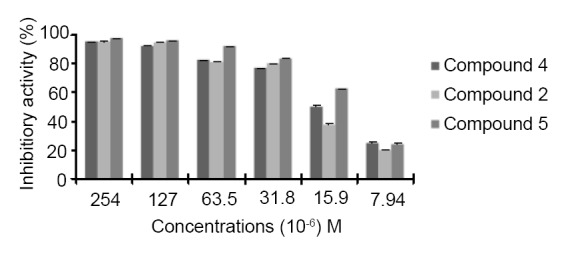
Antibacterial activity of compounds 2, 4 and 5 against *A.tumefaciens*.

As shown in Figure 6, all compounds are active against *A. tumefaciens*. Therefore, the organometallic compounds are more potent than their organic homologues. The highest inhibitory activity was observed with organometallic compound **4** and **5** at concentrations 15.9 × 10^-6^ M and 7.94 × 10^-6^ M. Moreover, the transformation to salts of citric acid preserves the same activity of our compounds with a slight increase (Figure 6).

### Tumor activity on Beet-Disc Assay

**Figure 7:**
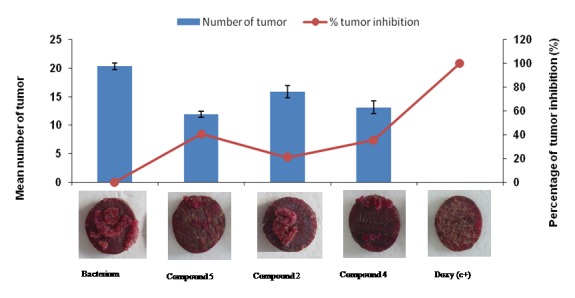
Percentage of tumor inhibition by compound 2, 4 and 5 on beet disc.

Different levels of crown gall tumor inhibition (20.7% to 40.55%) by the organic or organometallic compounds were observed on the beet discs, depending on the synthesized molecules against *A. tumefaciens* strain. Doxycycline (positive control) showed 100% tumor inhibition (Figure 7).

Organometallic compounds were able to produce significant tumor inhibition 35.48 % to 40.55 %, while the organic compound produced 20.7 %. The highest tumor inhibition was observed with compound **5** (40.55 %) followed by compound **4** (35.48%). It has been shown that ferrocenyl compounds are more active than their organic analogues against cancer cells (21)  and our results confirmed these results.

### Antiproliferative tests on hormone-independent breast cancer cells MDA-MB-231

**Figure 8:**
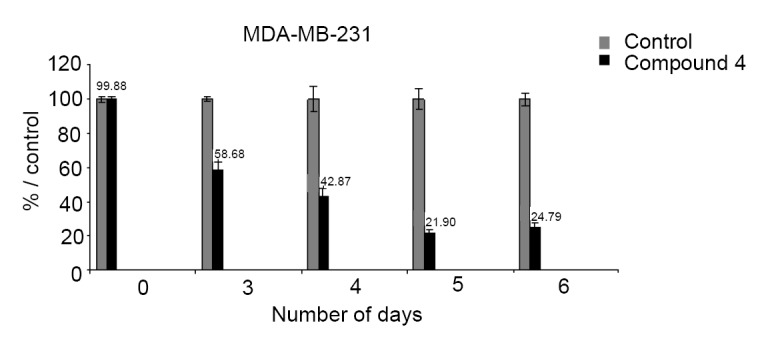
Effect of the compound 4 on the growth of hormone-independent breast cancer cells MDA-MB-231.

Antitumor activity was evaluated using the antiproliferative assay as described by Mehdi *et al.* (22). The compound **4** shows a strong antiproliferative effect on MDA-MB-231 with an IC_50_ of 0.45 µM. After 6 days of incubation, compound 4 showed 75% inhibition on the cell at low concentration 1 × 10^-6^. We have previously noted this effect on Beet-Disc Assay. We found significant crown gall inhibition 35.48% with this compound (considering more than 20% tumor inhibition is significant). As a consequence, the beet disc bioassay could be used effectively for screening different bioactive compounds in terms of antitumor activity (Figure 8).

## DISCUSSION

Bioassay tests have been playing an important role in the exploration of several clinically useful anti-cancer agents. Potato disc bioassay was based on *A. tumefaciens* infection on potato disc; it becomes useful for checking antitumor properties of biological and synthetic bioactive compounds. Coker *et al.* (9) examined several compounds known for their antitumor activity to be tested with the potato disc bioassay. This study was showing that camptothecin, palitaxel, podophyllin, vinblastine and vincristine have a significant inhibitory effect on the crown-gall tumor. As a consequence, the potato disc bioassay could be used effectively for screening different bioactive compounds in terms of antitumor activity. Nowadays several groups used potato disc bioassay for testing the antitumor properties of biological bioactive compounds (1-2, 16, 25-32). This assay has advantages of being rapid, inexpensive, simple and reliable pre-screen for antitumor activity. Meanwhile, potato disc method needs a lugol staining to display the crown gall formation. Many authors adopt other vegetables to test the antitumor activity such as carrot disc bioassay without any staining need (33). It became a necessity to compare several vegetables for selecting the best discs bioassay. In the present study, we found that the beet bioassay seems to be more adequate for the anti-tumor test screening. In fact, beet disc bioassay is more sensitive to crown gall disease with a fast expression of symptoms and more visible galls without any staining need.

Our selected beet disc bioassay allowed a significant crown gall tumor inhibition showing that the organic and organometallic compounds are a potential source of antitumor properties. This result is confirmed by testing compound **4** on MDA-MB-231 breast cancer cell line. Our results with the other compounds are in good agreement with previous findings dealing with significant antitumor activity of organic and organometallic compounds on hormone-dependent and hormone-independent breast cancer cells (21, 22).
